# *Porphyromonas gingivalis*-Derived Lipopolysaccharide Combines Hypoxia to Induce Caspase-1 Activation in Periodontitis

**DOI:** 10.3389/fcimb.2017.00474

**Published:** 2017-11-14

**Authors:** Ran Cheng, Wen Liu, Rui Zhang, Yuchao Feng, Neil A. Bhowmick, Tao Hu

**Affiliations:** ^1^State Key Laboratory of Oral Diseases & National Clinical Research Center for Oral Diseases & Department of Preventive Dentistry, West China Hospital of Stomatology, Sichuan University, Chengdu, China; ^2^Department of Medicine, Cedars-Sinai Medical Center, Los Angeles, CA, United States; ^3^State Key Laboratory of Oral Diseases & National Clinical Research Center for Oral Diseases & Department of Cariology and Endodontics, West China Hospital of Stomatology, Sichuan University, Chengdu, China

**Keywords:** *Porphyromonas gingivalis*, *Escherichia coli*, hypoxia, caspase-1, IL-1β

## Abstract

Periodontitis is defined as inflammation affecting the supporting tissue of teeth. Periodontal pathogens initiate the disease and induce inflammatory host response. Hypoxia may accelerate the process by producing pro-inflammatory factors. The aim of this study is to investigate the effect of *Porphyromonas gingivalis* (*P. gingivalis*) lipopolysaccharides (LPS) and *Escherichia coli* (*E. coli*) LPS in inducing caspase-1 activation in normoxic or hypoxic phases. The results showed that healthy gingiva was in a normoxic phase (HIF-1α negative). However, hypoxia appeared in periodontitis, in which NLRP3, cleaved-caspase-1, interleukin 1 beta (IL-1β) and caspase-1-induced cell death was enhanced in periodontitis specimens. The *in vitro* experiment showed that *P. gingivalis* LPS slightly decreased the level of NLRP3 and IL-1β in gingival fibroblasts under normoxia. Surprisingly, hypoxia reversed the effects of *P. gingivalis* LPS, highly promoted caspase-1 activation and IL-1β maturation. *E. coli* LPS, a kind of pathogen-associated molecular pattern (PAMP) was chosen to simulate the effect of Gram-negative microbiota. Different from *P. gingivalis* LPS, *E. coli* LPS enhanced IL-1β maturation both in normoxia and hypoxia. Moreover, *E. coli* LPS turned normoxia into hypoxia phase in experimental periodontitis model, which may subsequently propel the inflammatory effect of *P. gingivalis* LPS. It was concluded that *E. coli* LPS induced a hypoxic phase, which is a combing pathological factor of *P. gingivalis* LPS in caspase-1 activating and IL-1β maturation in periodontal inflammation.

## Introduction

Periodontitis is defined as chronic inflammation of tooth supporting tissues. Healthy periodontium maintain host-microbe homeostasis in which a controlled immuno-inflammatory state is kept. Imbalanced host-microbe interaction lead to disease initiation and progression (Cheng et al., 2015b). Several Gram-negative anaerobic and microaerophilic bacterial species, such as *Porphyromonas gingivalis, Treponema denticola, Tannerella forsythia*, and *Aggregatibacter actinomycetemcomitans* (*A. actinomycetemcomitans*) and the corresponding host responses, are the predominant etiological factors of periodontitis (Kinane, 2001). Pathological bacteria are significant inflammatory stimulus that triggers various cell types (epithelial cells, periodontal ligament fibroblasts, leukocytes, osteoblasts) to release pro-inflammatory factors, e.g., IL-1β, IL-6, tumor necrosis factor α (TNF-α), proteases, matrix metalloproteinases, prostaglandins (PGE), and so on Trindade et al. (2014). These inflammatory molecules lead to the breakdown of connective tissue and bone (Di Benedetto et al., 2013).

Hypoxia is a common feature of inflammation. Local inflammation disrupted microcirculation and induced leukocyte infiltration, disturbing the blood and oxygen supply (Karhausen et al., 2005). Periodontium may undergo a shift from normoxic phase to hypoxic phase when inflammation initiates and progresses. It has been estimated that the oxygen tension (pO_2_) at the base of untreated periodontal pockets is about 13.3 mm Hg (1.8% O_2_). The anaerobic environment provides a colonizing niche for Gram-negative anaerobes, whose growth would produce more metabolites and further lower the pO_2_ in deeper sites (Mettraux et al., 1984). Many studies also showed that hypoxia is a pathogenic factor in periodontitis. Hypoxia affects alveolar bone resorption by produce pro-inflammatory factors, IL-1β, IL-6, and PGE_2_ (Motohira et al., 2007). Hypoxia also influences the expression of receptor activator of nuclear factor kappa-B (NF-kB) ligand and osteoprotegerin, which accelerate the development of periodontitis (Yu et al., 2015). Thus, hypoxia is a propelling factor in periodontitis.

Pathological bacteria and hypoxia may activate caspase-1, a cysteine proteinase, which leads the activation and secretion of IL-1β, and IL-18. Activated-caspase-1 may also mediated pyroptosis, in which 1–2 nm plasma-membrane pores are formed, inducing potassium efflux, water influx, cell swelling, eventually osmotic lysis (Bergsbaken et al., 2009). We showed that caspase-1 activation and pyroptosis was involved in apical periodontitis (Cheng et al., 2017). In periodontitis, the periodontopathic pathogen, *A. actinomycetemcomitans*, has been involved in caspase-1 activation and secretion of IL-1β and IL-18 in the monocytes/macrophages (Johansson, 2011). Previous studies have shown that supragingival biofilm enhanced caspase-1 activity, IL-1β, and IL-18 gene expressions in gingival fibroblasts (Bostanci et al., 2011). However, a 10-specie subgingival biofilm model including *P. gingivalis* down-regulate NLRP3 and IL-1β expressions in gingival fibroblasts, partly because of *P. gingivalis* (Belibasakis et al., 2013). The findings seems controversial to the consensus that *P. gingivalis* is a keystone pathogen of periodontitis. Therefore, other factors, e.g., hypoxia was brought into consideration. The aim of this study is to investigate the effect of hypoxia on caspase-1 activation, IL-1β maturation, and a possible mechanism of hypoxia formation during periodontitis.

## Materials and methods

### Clinical specimens

This study was carried out in accordance with the recommendations of the Institutional Ethics Committee of West China Hospital of Stomatology with written informed consent from all subjects. All subjects gave written informed consent in accordance with the Declaration of Helsinki. The protocol was approved by the Institutional Ethics Committee of West China Hospital of Stomatology (WCHSIRB-ST-2014-091). Periodontitis tissues were obtained from patients (*n* = 5) diagnosed as chronic periodontitis undergoing gingivectomy. Healthy gingival tissues were harvested from patients (*n* = 3) without any periodontal diseases undergoing crown lengthening surgery. The included patients were diagnosed as chronic periodontitis in West China Hospital of Stomatology accordingly to classification of the periodontal diseases (Armitage, 1999). They had neither periodontal treatment nor antibiotic use for at least 3 months. The exclusion criteria included diabetes mellitus, pregnancy, liver, or kidney dysfunction, autoimmune diseases and infectious stomatitis (oral candidosis, herpetic stomatitis). The patient information was shown in Supplementary Table 1.

### Experimental periodontitis model

This study was carried out in accordance with the recommendations of the Guide for the Care and Use of Laboratory Animals from the National Institutes of Health. The protocol acquired the approval of the Institutional Animal Care and Use Committee, Cedars Sinai Medical Center (IACUC003638). The mice were housed at the Cedars Sinai Medical Center Animal Facility. 4–8 weeks old female Balb/c mice were obtained from the Harlan Laboratories (Madison, WI, USA). The experimental periodontitis model was established according to previous studies (Yokoyama et al., 2011; Cheng et al., 2015a). One hour before euthanization, the mice were injected into the peritoneal cavities with pimonidazole hydrochloride (Hypoxyprobe™-1; HPI, Inc., Burlington, MA, USA; Shi et al., 2013) at a dose of 60 mg/kg.

### Immunohistochemistry (IHC)

**Figure 1:**
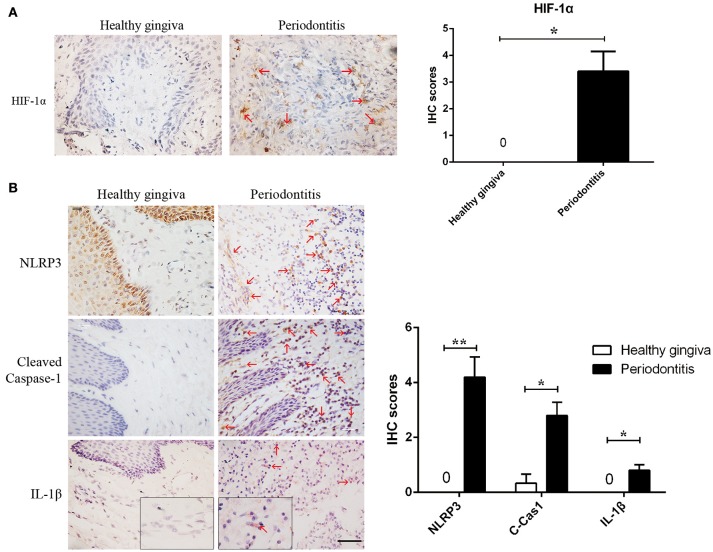
Periodontitis induced a hypoxic environment, in which caspase-1 was activated. **(A)** HIF-1α (red arrows) is positive in the gingival stroma of periodontitis specimens, while the healthy gingiva were negatively strained. (periodontitis specimens, *n* = 5; healthy gingiva, *n* = 3; ^*^P < 0.05; Bar, 50 μm) **(B)** NLRP3, cleaved-caspase-1 and IL-1β (red arrows) were increased in gingival stroma of periodontitis compared to the healthy control. (periodontitis specimens, *n* = 5; healthy gingiva, *n* = 3; ^*^P < 0.05; ^**^P < 0.01; C-Cas1, cleaved caspase-1; Cas1, caspase-1; Bar, 50 μm).

For human specimens, the IHC detection was conducted using antibodies anti-human HIF-1α (Abcam, Cambridge, MA, USA), anti-human NLRP3 (LifeSpan BioSciences, Seattle, WA, USA), anti-human cleaved-caspase-1 (Biorbyt Ltd., Cambridge, UK) and anti-human IL-1β (Abcam; mainly detect mature IL-1β). Images were captured by Nikon Eclipse 80i (Nikon Instruments Inc., Melville, NY, USA) and shown in Figure 1 and Supplementary Figure 1. For mouse tissues, the IHC was detected using anti-mouse NLRP3 (LifeSpan BioSciences, Seattle, WA, USA), anti-mouse IL-1β (R&D Systems Inc., Minneapolis, MN, USA; detect pro- and mature IL-1β). Images were captured by Aperio® AT2 (Leica Biosystems, Wetzlar, Germany), and shown in **Figure 4** and Supplementary Figure 2. The IHC scores were determined as described previously (Kreisberg et al., 2004).

### Quantum dot (QD) labeling and tunel dual labeling

The method was a modification of a previously described method (Lin et al., 2013). The antigen retrieved tissues incubated in the following sequences: the endogenous biotin-blocking kit (Molecular Probes Inc., Eugene, OR, USA) according to the instruction; PBS containing 2% bovine serum albumin (BSA) at 37°C for 30 min; equilibration buffer (KeyGEN.Co.Ltd., Nanjing, Jiangshu, China) for 10 min; the TdT reaction mix (rTdT enzyme and biotin-11-dUTP in equilibration buffer; KeyGEN. Co.Ltd.,) for 60 min; streptavidin-conjugated QD (QD-SA) 605 (Invitrogen, Carlsbad, CA, USA) for 30 min. After adequate wash, the specimens were incubated in 2% BSA at 37°C for 30 min; anti-human cleaved-caspase-1 (Biorbyt Ltd.,) for overnight at 4°C; 2% BSA at 37°C for 10 min; biotin-conjugated secondary antibody at 37°C for 30 min; 2% BSA at 37°C for 20 min; QD-SA 525 at 37°C for 30 min. Finally, the slides were mounted in 496-diamidino-2-phenylindole (DAPI) (Applygen Technologies Inc., Beijing, China) for 5 min. The images were taken by using a fluorescence microscope (Olympus Fsx100, Olympus Corporation, Tokyo, Japan).

### Cell culture

The human gingival tissue was cut into pieces, and then digested in 3 mg/mL collagenase type I (Hyclone, Logan, UT, USA) for 1 h at 37°C. Cells were cultured in Dulbecco's modified Eagle's medium (Gibco, Grand Island, NY, USA) adding 10% fetal calf serum (Biowest, France) plus 100 U/mL penicillin and 100 μg/mL streptomycin (Hyclone). Cells between passages 3–7 were used. Mouse gingival fibroblasts were cultured from gingiva obtained from 6 to 8 weeks old female Balb/c mice (Harlan Laboratories). The gingival tissue was cut and cultured in Alpha Modifications Minimum Essential Medium with 10% fetal bovine serum plus 100 U/mL penicillin and 100 μg/mL streptomycin (all from Cambrex, Walkersville, MD, USA) in a humidified atmosphere of 5% CO_2_ at 37°C. Cells between passages 3–6 were used.

### Western blot

Human gingival fibroblasts were serum-starved for 24 h then treated with 1 μg/mL *E. coli* LPS [*Escherichia coli* LPS (O111:B4; Sigma-aldrich, St Louis, MO, USA)] (Herath et al., 2011) or 10 μg/mL *P. gingivalis* LPS (Invivogen, San Diego, CA, USA) (Abe-Yutori et al., 2017) at 2 or 20% O_2_ for 6 h. Mouse gingival fibroblasts were serum-starved for 24 h then were treated with 1 μg/mL *E. coli* LPS at 2% O_2_ for 1, 2, 4, and 24 h. Cells were collected and proteins were extracted in lysis buffer (Keygen Biotech Inc., Nanjing, China) and 20–30 μg of the total proteins were loaded and separated by using 10% SDS-PAGE. The following proteins were detected using antibodies, anti-human, -mouse NLRP3 (LifeSpan BioSciences, Seattle, WA, USA); anti-human, -mouse caspase-1 (Santa Cruz Biotechnology, Inc., Santa Cruz, CA, USA); anti-human IL-1β (Abcam; detect pro- and mature IL-1β) and anti-mouse IL-1β (Novus Biologicals, Inc., Littleton, CO, USA; detect pro- and mature IL-1β) were used. The proteins were visualized using an enhanced chemiluminescence kit (Millipore Inc., Darmstadt, Germany). The bands were analyzed by using Quantity One (Bio-Rad).

### RT-PCR

Mouse gingival fibroblasts were serum-starved for 24 h then stimulated with 1 μg/mL *E. coli* LPS for 24 h. Total mRNA was extracted using RNase mini kit (Giagen, Hilden, Germany). The total RNA were reversely transcript to cDNA by (Bio-Rad Laboratories, Inc., Hercules, CA, USA). The mRNA expression of IL-1β was measured by RT-PCR, which was performed on Bio-radS1000™ Thermal Cycler (Bio-Rad Laboratories) using GoTaq Green Master Mix (VWR International, Radnor, PA, USA). Primer sequences were forward ACCTAGCTGTCAACGTGTGG; reverse TCAAAGCAATGTGCTGGTGC.

### Statistics

Data were expressed as mean ± SD. The statistical significance of differences among groups was assessed using Student's *t*-test or one-way ANOVA by SPSS 16.0 (IBM Corp. New York, NY, USA). A difference was considered significant if *P* < 0.05.

## Results

### Hypoxia and caspase-1 activation exist in periodontitis

**Figure 2:**
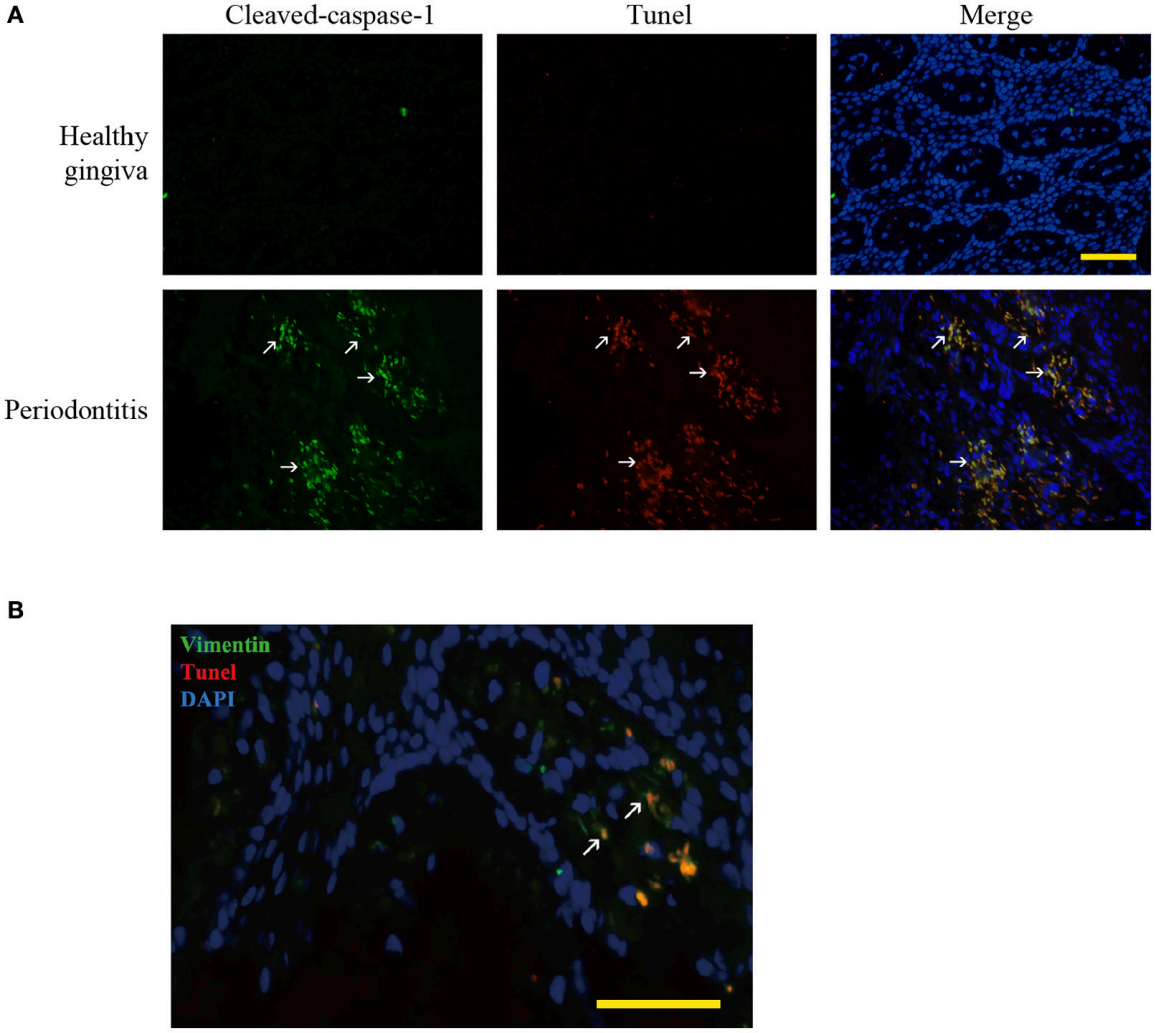
Pyroptosis in periodontitis. **(A)** Dual labeling of cleaved-caspase-1 and Tunel showed that the stains were overlapped. It verified that caspase-1 mediated the cell death, pyroptosis (white arrow) in periodontitis. (Bar, 50 μm). **(B)** Dual labeling of vimentin and Tunel in periodontitis. It showed that the stains co-existed in many cells (white arrow), showing that gingival fibroblasts suffered from cell death in periodontitis. (Bar, 50 μm).

We found hypoxia to be common in human periodontitis afflicted gingival tissues, as evidence by the expression of the transcription factor, HIF-1α (hypoxia-inducible factor α) (Ng et al., 2011; Figure 1A). Conversely, healthy gingiva presented a negative staining of HIF-1α, suggesting that periodontal inflammation induced a shift from normoxic phase to hypoxic phase. As hypoxia and elevated reactive oxygen are associated with inflammasome activation, we tested its presence in patient gingiva. In response to bacterial infection, the NLRP3 inflammasome would be assembled for the caspase-1 activation. The subsequent activation of caspase-1 promotes the release of the proinflammatory cytokine IL-1β in contributing to the inflammatory response (Kim and Jo, 2013). In this study, we examined the expressions of NLRP3, cleaved-caspase-1 (active), and IL-1β expression, each of which were upregulated in human periodontitis gingival tissue, compared to health gingiva (Figure 1B). In support of caspase-1-mediated cell death, pyroptosis, we found co-expressions of cleaved-caspase-1 and Tunel in the periodontitis tissues. The co-staining supported that activated caspase-1 was associated with cell death (Figure 2A). Gingival fibroblasts also suffered from cell death in periodontitis (Figure 2B). The hypoxic environment, usually accompanying inflammation, is a potential pathological factor in itself (Eltzschig and Carmeliet, 2011). Although clinical evidence and research support *P. gingivalis* as a key periodontal pathogen, we speculated that hypoxia can be involved in periodontal cell death process.

### The synergistic effects of LPS and hypoxia activated caspase cascade in human gingival fibroblasts

There is a longstanding paradox that *P. gingivalis* LPS reveals low inflammatory potency though it is the “keystone pathogen.” The paradox might be explained by the synergistic infection of *P. gingivalis* and commensal microbial community (Darveau et al., 2012). LPS is the principal component of Gram-negative bacteria that activates the innate immune system. Here *E. coli* LPS, a kind of PAMP, was used to study the effect of Gram-negative microbiota. Even though *E. coli* LPS does not come from oral bacteria, it has been used in stimulating *in vivo* and *in vitro* periodontal inflammation (Gürkan et al., 2009; Lu et al., 2017). We also simulated normoxic and hypoxic conditions to study the effects of two kinds of LPS *in vitro*.

**Figure 3:**
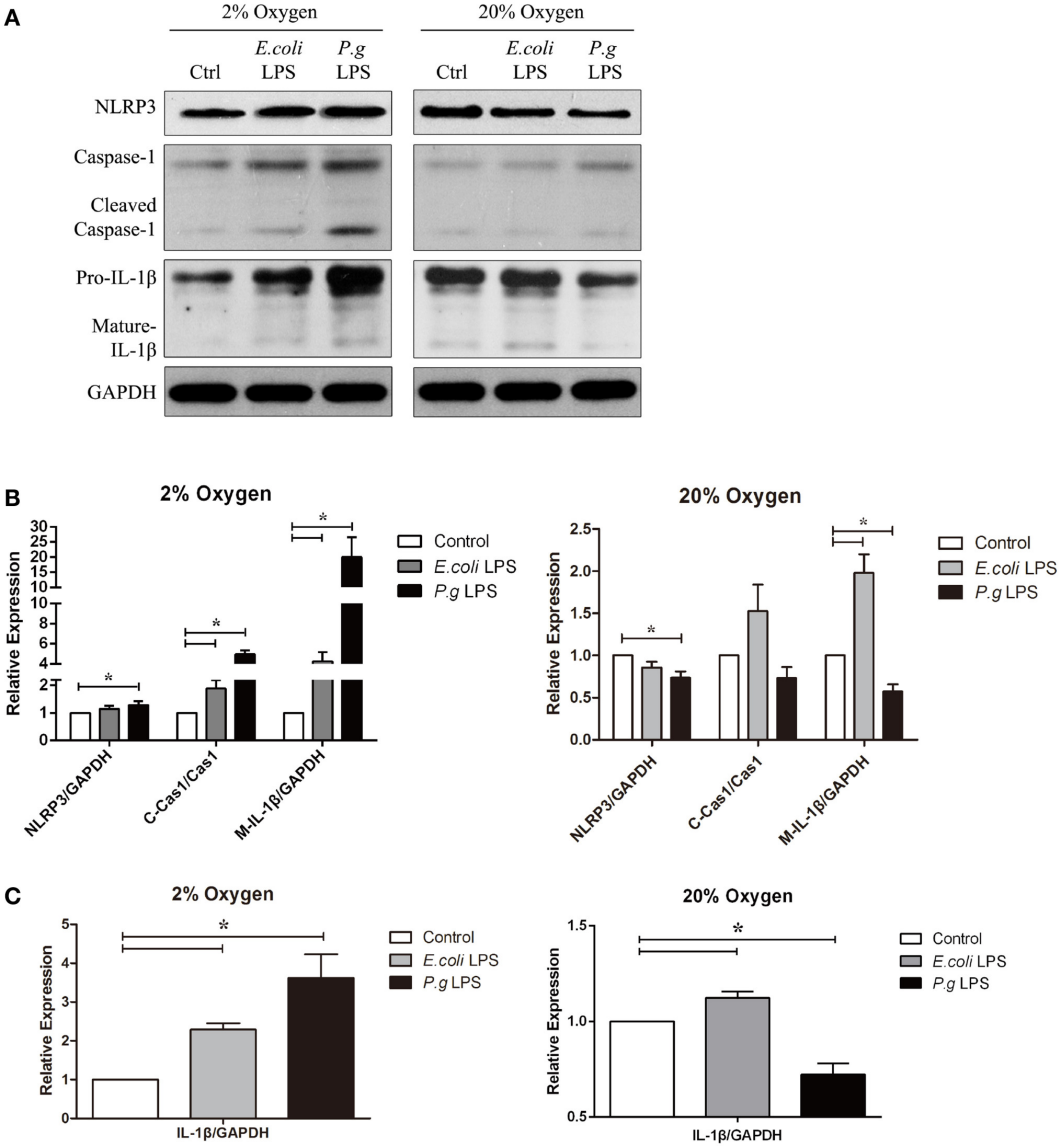
Hypoxia and LPS increased pro-IL-1β, caspase-1 activation and IL-1β maturation *in vitro*. **(A)** Human gingival fibroblasts were stimulated with *E. coli* LPS or *P. gingivalis* LPS at 2 or 20% O_2_ for 6 h. The protein levels of NLRP3, caspase-1 and IL-1β were detected by western blot. (Ctrl, control; *P.g*., *P. gingivalis*) **(B,C)** Densitometric analyses were shown. *E. coli* LPS increased the pro-IL-1β, in both hypoxic and normoxic conditions. However, *P. gingivalis* LPS had two-side effects in the production of pro-IL-1β. In normoxic condition, *P. gingivalis* LPS had decreased pro-IL-1β production. But the effects were reversed when hypoxia were combined *P. gingivalis* LPS decreased NLRP3 and IL-1β expressions under normoxia, but had reverse results in hypoxic environment. Hypoxia also promoted the caspase-1 activation and maturation of IL-1β in combination of *E. coli* LPS. (*n* = 3; ^*^P < 0.05; C-Cas1, cleaved caspase-1; Cas1, caspase-1; M-IL-1β, mature IL-1β).

Our data showed that *P. gingivalis* LPS down-regulated NLRP3, IL-1β precursor (pro-IL-1β) and mature IL-1β under normoxia. However, under moderate hypoxia (2% O_2_), *P. gingivalis* LPS enhanced NLRP3 expression, cleaved-caspase-1, and mature IL-1β (Figure 2). Further, the protein level of pro-IL-1β was increased (Figure 2), suggesting a NF-kB transcriptional activity. The *E. coli* LPS induced pro-IL-1β expression and maturation of IL-1β, which was associated with cleaved-caspase-1, under hypoxic condition. But mature IL-1β was also upregulated compared to control under normoxic condition (Figure 3). Therefore, *E. coli* LPS was able to increase mature IL-1β in both normoxic and hypoxic phases. *E. coli* LPS-induced inflammation would be a candidate reason in the shift from normoxic phase to hypoxic phase.

### *E. coli* LPS induced hypoxia and Il-1β production in experimental model of periodontitis

To verify the assumption that *E. coli* LPS may induce hypoxia, *E. coli* LPS-induced periodontitis model was chosen in this experiment. We found evidence of hypoxia in the periodontitis model, as determined by hypoxyprobe localization. The results showed that *E. coli* LPS was capable of changing normoxic phase (the control) to hypoxic phase (the periodontitis model) *in vivo*. The results also suggested that Gram-negative microbiota may prepare a favorable environment for *P. gingivalis* and helped to propel caspase-1 activation.

**Figure 4:**
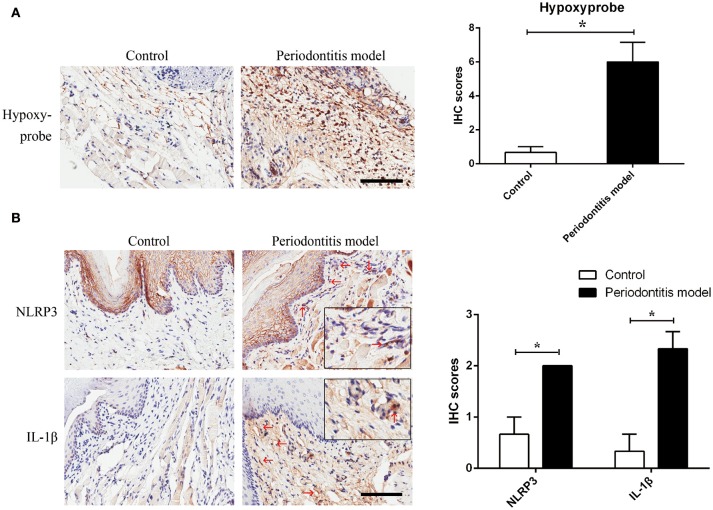
Experimental periodontitis, generated by the injection of *E. coli* LPS, induced a chronic hypoxic environment, in which NLRP3 and IL-1β were enhanced. **(A)** Hypoxyprobe visualized a hypoxic environment in the stroma of gingival tissue. Experimental periodontitis model showed positive staining of hypoxyprobe. Application of anakinra partly alleviated the hypoxic condition. (*n* = 3; ^*^P < 0.05) **(B)** NLRP3 and IL-1β were increased in gingival stroma of the experimental periodontitis model compared to the control. (*n* = 3–4; Bar, 100 μm; ^*^P < 0.05).

**Figure 5:**
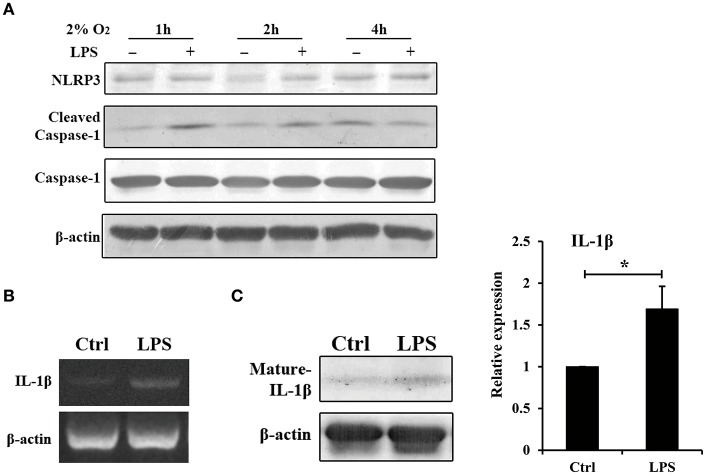
Hypoxia and *E. coli* LPS increased IL-1β production in mouse gingival fibroblasts *in vitro*. **(A)** Mouse gingival fibroblasts were stimulated with *E. coli* LPS at 2% O_2_ for 1, 2, and 4 h. NLRP3 and caspase-1 were examined by western blot. Compared to the hypoxia negative control, cleaved-caspase-1 was increased by LPS at 1 and 2 h, but decreased at 4 h. **(B)** Mouse gingival fibroblasts were stimulated with *E. coli* LPS at 2% O_2_ for 24 h. The mRNA level of IL-1β was examined by RT-PCR. The expression of pro-IL-1β was enhanced by LPS at 24 h. **(C)** After stimulated with *E. coli* LPS for 24 h at 2% O_2_, mouse gingival fibroblasts had higher level of mature IL-1β. Densitometric analyses were shown. (*n* = 3; ^*^P < 0.05).

In the experimental periodontitis model, NLRP3 and IL-1β were also enhanced, similar to the results of human periodontitis specimen (Figure 4B). The mechanism of pro-IL-1β and mature IL-1β generation was tested in mouse gingival fibroblasts with *E. coli* LPS at 2% O_2_. We found that NLRP3 up regulation at 2 h of treatment was short lived (Figure 5A). Yet, cleaved-caspase-1 was enhanced by hypoxia and *E. coli* LPS at 1 h and 2 h. But by 4 h, *E. coli* LPS had little effect on either NLRP3 or cleaved caspase-1 expression. *E. coli* LPS were associated with elevated IL-1β transcriptional expression. Caspase-1 activation was the mechanism of mature IL-1β production in mouse gingival fibroblasts (Figures 5B,C). Together, the results in mouse *in vivo* and *in vitro* models confirmed that *E. coli* LPS induced hypoxic phase, which subsequently participated in the caspase-1 activation and mature IL-1β generation.

## Discussion

### Caspase-1 was engaged in the inflammatory process of periodontitis

When infection initiates, NOD-like receptors (NLRs) are able to assemble a multiprotein complex (inflammasome), which activated caspase-1 and even induced pyroptotic (Vande Walle and Lamkanfi, 2016). Some studies have provided clues that inflammasome and caspase-1 be involved in the initiation and progression of periodontitis. For example, much higher NLRP3 inflammasome components were detected in periodontitis tissues than from healthy gingiva (Huang et al., 2015; Xue et al., 2015). The NLRP3 inflammasome increased the secretion of IL-1β, IL-6, and TNF-α in human periodontal ligament fibroblasts (Lu et al., 2017). Our study verified that NLRP3, cleaved-caspase-1 and IL-1β were enhanced in periodontitis tissues (Figure 1). Caspase-1 activation finally led to pyroptotic cell death (Figure 2). Usually, caspase-1 activation function as a host defense mechanism in eliminating pathogens (Yang et al., 2015). Caspase-1 induced human leukocytes lysis and IL-1β secretion under the attack of leukotoxin produced by *A. actinomycetemcomitans* (Kelk et al., 2008, 2011). However, elevated or inappropriate caspase-1 activation can lead to inflammation and excessive cell death (Simon and van der Meer, 2007). Caspase-1 is involved in the pathogenesis of inflammatory diseases, including inflammatory bowel disease, neurodegenerative diseases, and endotoxic shock (Bergsbaken et al., 2009). It is therefore reasonable to consider caspase-1 be involved in the pathogenesis of periodontitis.

### Hypoxia was indispensable in *P. gingivalis*-induced caspase-1 activation

Hypoxia is a key factor of propelling caspase-1 activation. In macrophages, hypoxia increased the NLRP3, caspase-1 activation and mature IL-1β secretion (Folco et al., 2014). In hepatocellular carcinoma cells, hypoxia induces caspase-1 activation, cleavage and release of proinflammatory cytokines, IL-1β and IL-18 (Yan et al., 2012). Our data for the first time demonstrated the importance of hypoxia in the mechanism of caspase-1 activation and IL-1β maturation. The effect of *E. coli* LPS was enhanced by hypoxia on gingival fibroblasts. Furthermore, hypoxia reversed the effect of *P. gingivalis* on NLRP3, caspase-1 activation and production of mature IL-1β *in vitro*. Unlike IL-6 and TNF-α, IL-1β has a unique post-translational modification and secretion mechanism. IL-1β is produced as a proprotein, which is proteolysed to its active form by caspase 1. Then the plasma-membrane pores produced by pyroptosis are helpful for the secretion (Piccioli and Rubartelli, 2013). It suggested that caspase-1 and pyroptosis is important in producing and releasing IL-1β in periodontitis. However, the secretory IL-1β was very low in this study, suggesting that the secretory process requires further investigation (Supplementary Figure 3).

### *E. coli* LPS helped to produce hypoxia

Therefore, the shift from normoxic phase to hypoxic phase may be a potential milestone in periodontitis, from which the inflammation would be exaggerated. The Gram-negative microbiota, as we assumed, could influence the phase conversion. In this study, *E. coli* LPS successfully induced a hypoxic environment in the experimental periodontitis model. As a facultative anaerobe, *E. coli* LPS induced the caspase-1 cascade under normoxia, which might function as a defense mechanism in eliminating pathogens. However, *E. coli* LPS also increased IL-1β and thus induced hypoxia in the gingiva in a chronic process.

### Hypoxia and *P. gingivalis* LPS has synergistic effects in periodontitis

As an anaerobe, *P. gingivalis* scarcely survives under normorxia. Correspondingly, hypoxic condition could be a more favorable environment for *P. gingivalis*. This pattern of oxygen tension should accompany bacterial toxin in mediating inflammatory responses. Previous studies showed a paradox that *P. gingivalis* was strongly associated with periodontitis but was not an apparent inducer of inflammation (Darveau et al., 2012). *P. gingivalis* LPS revealed an unusually low inflammatory effect compared with *E. coli* LPS and LPS from many other Gram-negative bacteria (Munford and Varley, 2006). The environment factor, hypoxia, was a reasonable explanation for the paradox. It was established that *P. gingivalis*-induced periodontitis requires the presence of the commensal microbiota (Hajishengallis et al., 2011). A model of 11 subgingival biofilm (including *P. gingivalis*) and gingival tissues also up-regulated IL-1β secretion under hypoxia (Bao et al., 2015). Now we replenish that the effect of *E. coli* LPS (represent LPS from Gram-negative bacteria) helps to prepare a hypoxic environment, which exaggerate the inflammatory effect of *P. gingivalis*. The assumption may be further verified by using germ-free mice, which supplies an ideal model to study the synergistic effects of *E. coli* LPS and *P. gingivalis* LPS.

## Conclusion

In periodontitis, hypoxia is a propelling factor of *P. gingivalis* LPS in activating caspase-1 cascade. *E. coli* LPS induced the shift from normoxic phase to hypoxic phase. The study provided a type of interaction between hypoxia and LPS, by which *P. gingivalis* could function as “keystone pathogen.”

## Authors contributions

TH and NB designed the experiment. RC, WL, YF, and RZ executed the experiment. RC acquired and analyzed the data. RC wrote the manuscript. TH and NB made critical revision. All the authors give final approval and agree to be accountable for all aspects of the work.

### Conflict of interest statement

The authors declare that the research was conducted in the absence of any commercial or financial relationships that could be construed as a potential conflict of interest.

## Supplementary material

Supplementary Figure 1 The IHC staining of negative control, HIF-1α, NLRP3, cleaved-caspase-1 and IL-1β in other clinical samples were shown.

Supplementary Figure 2 The IHC staining of negative control, Hypoxyprobe, NLRP3 and IL-1β in other experimental models were shown.

Supplementary Figure 3 The secretory IL-1β in cell supernatant at 24h were measured by ELISA. There was no difference among groups.

## References

[B1] Abe-Yutori M. Chikazawa T. Shibasaki K. Murakami S. (2017). Decreased expression of E-cadherin by *Porphyromonas gingivalis*-lipopolysaccharide attenuates epithelial barrier function. J. Periodont. Res. 52, 42–50. 10.1111/jre.12367 27016120

[B2] Armitage G. C. (1999). Development of a classification system for periodontal diseases and conditions. Ann. Periodontol. 4, 1–6. 10.1902/annals.1999.4.1.1 10863370

[B3] Bao K. Papadimitropoulos A. Akgül B. Belibasakis G. N. Bostanci N. (2015). Establishment of an oral infection model resembling the periodontal pocket in a perfusion bioreactor system. Virulence 6, 265–273. 10.4161/21505594.2014.978721 25587671 PMC4601317

[B4] Belibasakis G. N. Guggenheim B. Bostanci N. (2013). Down-regulation of NLRP3 inflammasome in gingival fibroblasts by subgingival biofilms: involvement of *Porphyromonas gingivalis*. Innate Immun. 19, 3–9. 10.1177/1753425912444767 22522430

[B5] Bergsbaken T. Fink S. L. Cookson B. T. (2009). Pyroptosis: host cell death and inflammation. Nat. Rev. Microbiol. 7, 99–109. 10.1038/nrmicro2070 19148178 PMC2910423

[B6] Bostanci N. Meier A. Guggenheim B. Belibasakis G. N. (2011). Regulation of NLRP3 and AIM2 inflammasome gene expression levels in gingival fibroblasts by oral biofilms. Cell. Immunol. 270, 88–93. 10.1016/j.cellimm.2011.04.002 21550598

[B7] Cheng R. Choudhury D. Liu C. Billet S. Hu T. Bhowmick N. A. (2015a). Gingival fibroblasts resist apoptosis in response to oxidative stress in a model of periodontal diseases. Cell Death Discov. 1:15046. 10.1038/cddiscovery.2015.46 27551475 PMC4979524

[B8] Cheng R. Feng Y. Zhang R. Liu W. Lei L. Hu T. (2017). The extent of pyroptosis varies in different stages of apical periodontitis. BBA-Mol. Basis Dis. 1864, 226–237. 10.1016/j.bbadis.2017.10.025 29066283

[B9] Cheng R. Hu T. Bhowmick N. A. (2015b). Be resistant to apoptosis: a host factor from gingival fibroblasts. Cell Death Dis. 6:e2009. 10.1038/cddis.2015.350 26633715 PMC4720885

[B10] Darveau R. P. Hajishengallis G. Curtis M. A. (2012). *Porphyromonas gingivalis* as a potential community activist for disease. J. Dent. Res. 91, 816–820. 10.1177/0022034512453589 22772362 PMC3420389

[B11] Di Benedetto A. Gigante I. Colucci S. Grano M. (2013). Periodontal disease: linking the primary inflammation to bone loss. Clin. Dev. Immunol. 2013:503754. 10.1155/2013/503754 23762091 PMC3676984

[B12] Eltzschig H. K. Carmeliet P. (2011). Hypoxia and inflammation. N. Engl. J. Med. 364, 656–665. 10.1056/NEJMra0910283 21323543 PMC3930928

[B13] Folco E. J. Sukhova G. K. Quillard T. Libby P. (2014). Moderate hypoxia potentiates interleukin-1β production in activated human macrophages. Circ. Res. 115, 875–883. 10.1161/CIRCRESAHA.115.304437 25185259 PMC4209192

[B14] Gürkan A. Emingil G. Nizam N. Doganavşargil B. Sezak M. Kütükçüler N.. (2009). Therapeutic efficacy of vasoactive intestinal peptide in *Escherichia coli* lipopolysaccharide-induced experimental periodontitis in rats. J. Periodontol. 80, 1655–1664. 10.1902/jop.2009.090031 19792856

[B15] Hajishengallis G. Liang S. Payne M. A. Hashim A. Jotwani R. Eskan M. A.. (2011). Low-abundance biofilm species orchestrates inflammatory periodontal disease through the commensal microbiota and complement. Cell Host Microbe. 10, 497–506. 10.1016/j.chom.2011.10.006 22036469 PMC3221781

[B16] Herath T. D. Wang Y. Seneviratne C. J. Lu Q. Darveau R. P. Wang C. Y.. (2011). *Porphyromonas gingivalis* lipopolysaccharide lipid A heterogeneity differentially modulates the expression of IL-6 and IL-8 in human gingival fibroblasts. J. Clin. Periodontol. 38, 694–701. 10.1111/j.1600-051X.2011.01741.x 21752043

[B17] Huang X. Yang X. Ni J. Xie B. Liu Y. Xuan D.. (2015). Hyperglucose contributes to periodontitis: involvement of the NLRP3 pathway by engaging the innate immunity of oral gingival epithelium. J. Periodontol. 86, 327–335. 10.1902/jop.2014.140403 25325516

[B18] Johansson A. (2011). Aggregatibacter actinomycetemcomitans leukotoxin: a powerful tool with capacity to cause imbalance in the host inflammatory response. Toxins (Basel) 3, 242–259. 10.3390/toxins3030242 22069708 PMC3202821

[B19] Karhausen J. Haase V. H. Colgan S. P. (2005). Inflammatory hypoxia: role of hypoxia-inducible factor. Cell Cycle 4, 256–258. 10.4161/cc.4.2.1407 15655360

[B20] Kelk P. Abd H. Claesson R. Sandström G. Sjöstedt A. Johansson A. (2011). Cellular and molecular response of human macrophages exposed to *Aggregatibacte*r actinomycetemcomitans leukotoxin. Cell Death Dis. 2:e126. 10.1038/cddis.2011.6 21390060 PMC3101819

[B21] Kelk P. Claesson R. Chen C. Sjöstedt A. Johansson A. (2008). IL-1β secretion induced by *Aggregatibacter* (*Actinobacillus*) actinomycetemcomitans is mainly caused by the leukotoxin. Int. J. Med. Microbiol. 298, 529–541. 10.1016/j.ijmm.2007.06.005 17888725

[B22] Kim J. J. Jo E. K. (2013). NLRP3 inflammasome and host protection against bacterial infection. J. Korean Med. Sci. 28, 1415–1423. 10.3346/jkms.2013.28.10.1415 24133343 PMC3792593

[B23] Kinane D. F. (2001). Causation and pathogenesis of periodontal disease. Periodontol. 2000 25, 8–20. 10.1034/j.1600-0757.2001.22250102.x 11155179

[B24] Kreisberg J. I. Malik S. N. Prihoda T. J. Bedolla R. G. Troyer D. A. Kreisberg S.. (2004). Phosphorylation of Akt (Ser473) is anexcellent predictor of poor clinical outcome in prostate cancer. Cancer Res. 64, 5232–5236. 10.1158/0008-5472.CAN-04-0272 15289328

[B25] Lin J. Shou X. Mao X. Dong J. Mohabeer N. Kushwaha K. K.. (2013). Oxidized low density lipoprotein induced caspase-1 mediated pyroptotic cell death in macrophages: implication in lesion instability? PLoS ONE 8:e62148. 10.1371/journal.pone.0062148 23637985 PMC3636212

[B26] Lu W. L. Song D. Z. Yue J. L. Wang T. T. Zhou X. D. Zhang P.. (2017). NLRP3 inflammasome may regulate inflammatory response of human periodontal ligament fibroblasts in an apoptosis-associated speck-like protein containing a CARD (ASC)-dependent manner. Int. Endod. J. 50, 967–975. 10.1111/iej.12722 27864974

[B27] Mettraux G. R. Gusberti F. A. Graf H. (1984). Oxygen tension pO_2_ in untreated human periodontal pockets. J. Periodontol. 55, 516–521. 10.1902/jop.1984.55.9.516 6592325

[B28] Motohira H. Hayashi J. Tatsumi J. Tajima M. Sakagami H. Shin K. (2007). Hypoxia and reoxygenation augment bone-resorbing factor production from human periodontal ligament cells. J. Periodontol. 78, 1803–1809. 10.1902/jop.2007.060519 17760552

[B29] Munford R. S. Varley A. W. (2006). Shield as signal: lipopolysaccharides and the evolution of immunity to Gram-negative bacteria. PLoS. Pathog. 2:e67. 10.1371/journal.ppat.0020067 16846256 PMC1483240

[B30] Ng K. T. Li J. P. Ng K. M. Tipoe G. L. Leung W. K. Fung M. L. (2011). Expression of hypoxia-inducible factor-1α in human periodontal tissue. J. Periodontol. 82, 136–141. 10.1902/jop.2010.100100 21043802

[B31] Piccioli P. Rubartelli A. (2013). The secretion of IL-1β and options for release. Semin. Immunol. 25, 425–429. 10.1016/j.smim.2013.10.007 24201029

[B32] Shi Y. Oeh J. Eastham-Anderson J. Yee S. Finkle D. Peale F. V. Jr.. (2013). Mapping *in vivo* tumor oxygenation within viable tumor by 19F-MRI and multispectral analysis. Neoplasia 15, 1241–1250. 10.1593/neo.131468 24339736 PMC3858898

[B33] Simon A. van der Meer J. W. (2007). Pathogenesis of familial periodic fever syndromes or hereditary autoinflammatory syndromes. Am. J. Physiol. Regul. Integr. Comp. Physiol. 292, R86–R98. 10.1152/ajpregu.00504.2006 16931648

[B34] Trindade F. Oppenheim F. G. Helmerhorst E. J. Amado F. Gomes P. S. Vitorino R. (2014). Uncovering the molecular networks in periodontitis. Proteomics Clin. Appl. 8, 748–761. 10.1002/prca.201400028 24828325 PMC4426160

[B35] Vande Walle L. Lamkanfi M. (2016). Pyroptosis. Curr. Biol. 26, R568–R572. 10.1016/j.cub.2016.02.019 27404251

[B36] Xue F. Shu R. Xie Y. (2015). The expression of NLRP3, NLRP1 and AIM2 in the gingival tissue of periodontitis patients: RT-PCR study and immunohistochemistry. Arch. Oral Biol. 60, 948–958. 10.1016/j.archoralbio.2015.03.005 25841070

[B37] Yan W. Chang Y. Liang X. Cardinal J. S. Huang H. Thorne S. H.. (2012). High-mobility group box 1 activates caspase-1 and promotes hepatocellular carcinoma invasiveness and metastases. Hepatology 55, 1863–1875. 10.1002/hep.25572 22234969 PMC4610360

[B38] Yang Y. Jiang G. Zhang P. Fan J. (2015). Programmed cell death and its role in inflammation. Mil. Med. Res. 19, 12 10.1186/s40779-015-0039-0 PMC4455968 26045969

[B39] Yokoyama M. Ukai T. Ayon Haro E. R. Kishimoto T. Yoshinaga Y. Hara Y. (2011). Membrane-bound CD40 ligand on T cells from mice injected with lipopolysaccharide accelerates lipopolysaccharide-induced osteoclastogenesis. J. Periodontal. Res. 46, 464–474. 10.1111/j.1600-0765.2011.01362.x 21521224

[B40] Yu X. J. Xiao C. J. Du Y. M. Liu S. Du Y. Li S. (2015). Effect of hypoxia on the expression of RANKL/OPG in human periodontal ligament cells *in vitro*. Int. J. Clin. Exp. Pathol. 8, 12929–12935. 26722486 PMC4680431

