# Supplementary figures and images for: Porphyromonas gingivalis-Derived Lipopolysaccharide Combines Hypoxia to Induce Caspase-1 Activation in Periodontitis
Source: Front Cell Infect Microbiol. 2017 Nov 14;7:474. doi: 10.3389/fcimb.2017.00474 (PMC5694474; doi:10.3389/fcimb.2017.00474)

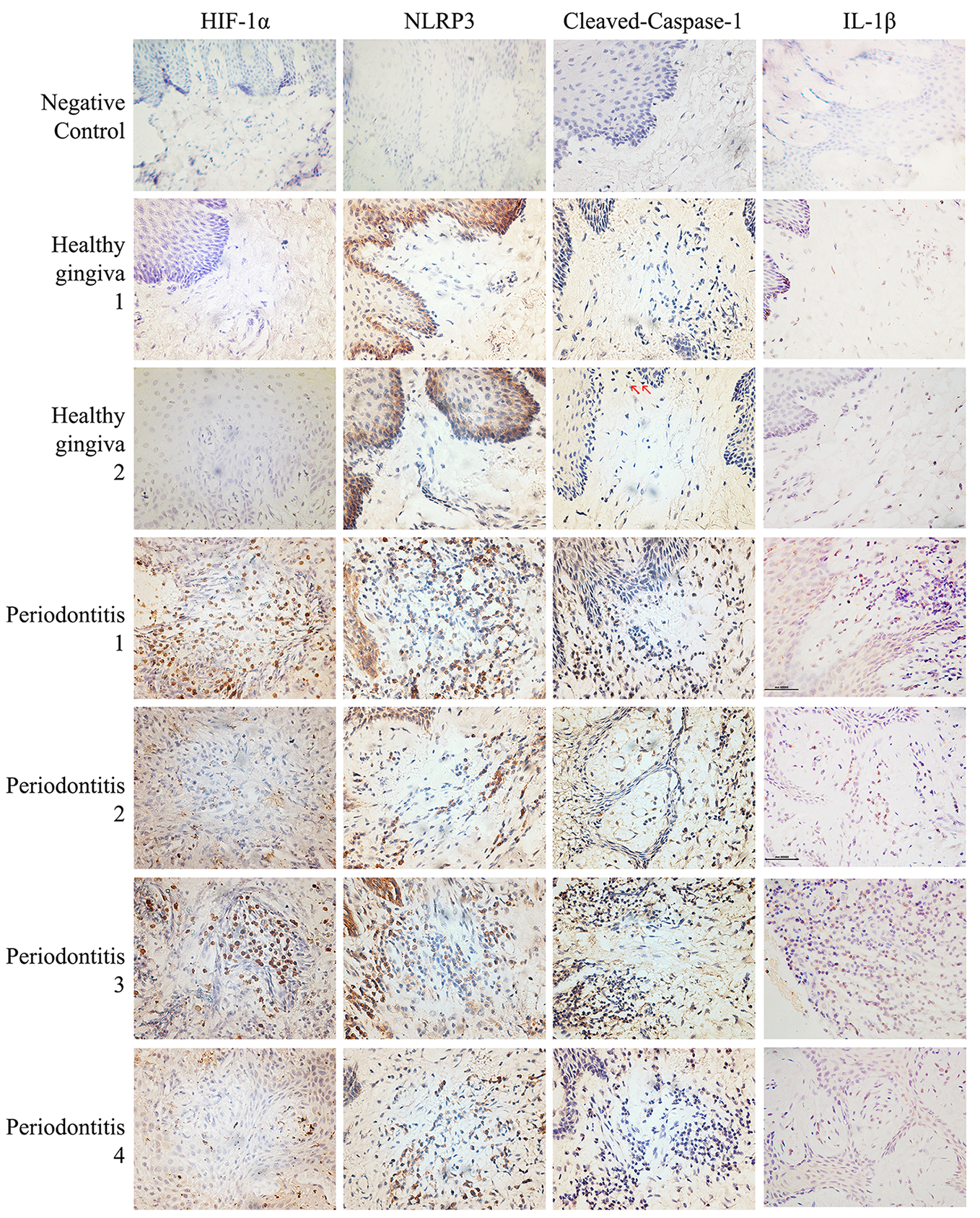

Supplement: Supplementary Figure 1 — The IHC staining of negative control, HIF-1α, NLRP3, cleaved-caspase-1 and IL-1β in other clinical samples were shown. [file Image1.TIF]

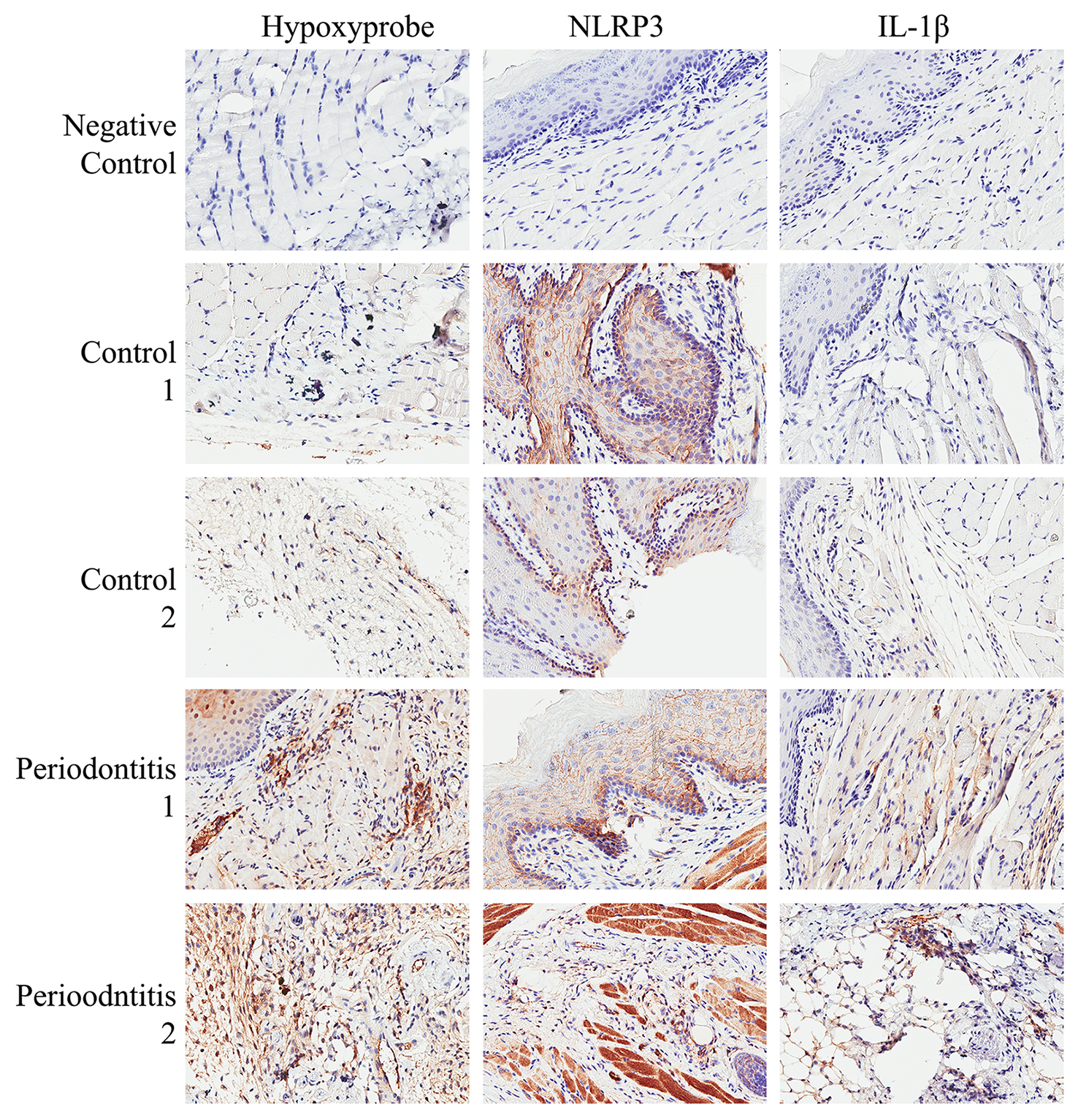

Supplement: Supplementary Figure 2 — The IHC staining of negative control, Hypoxyprobe, NLRP3 and IL-1β in other experimental models were shown. [file Image2.TIF]

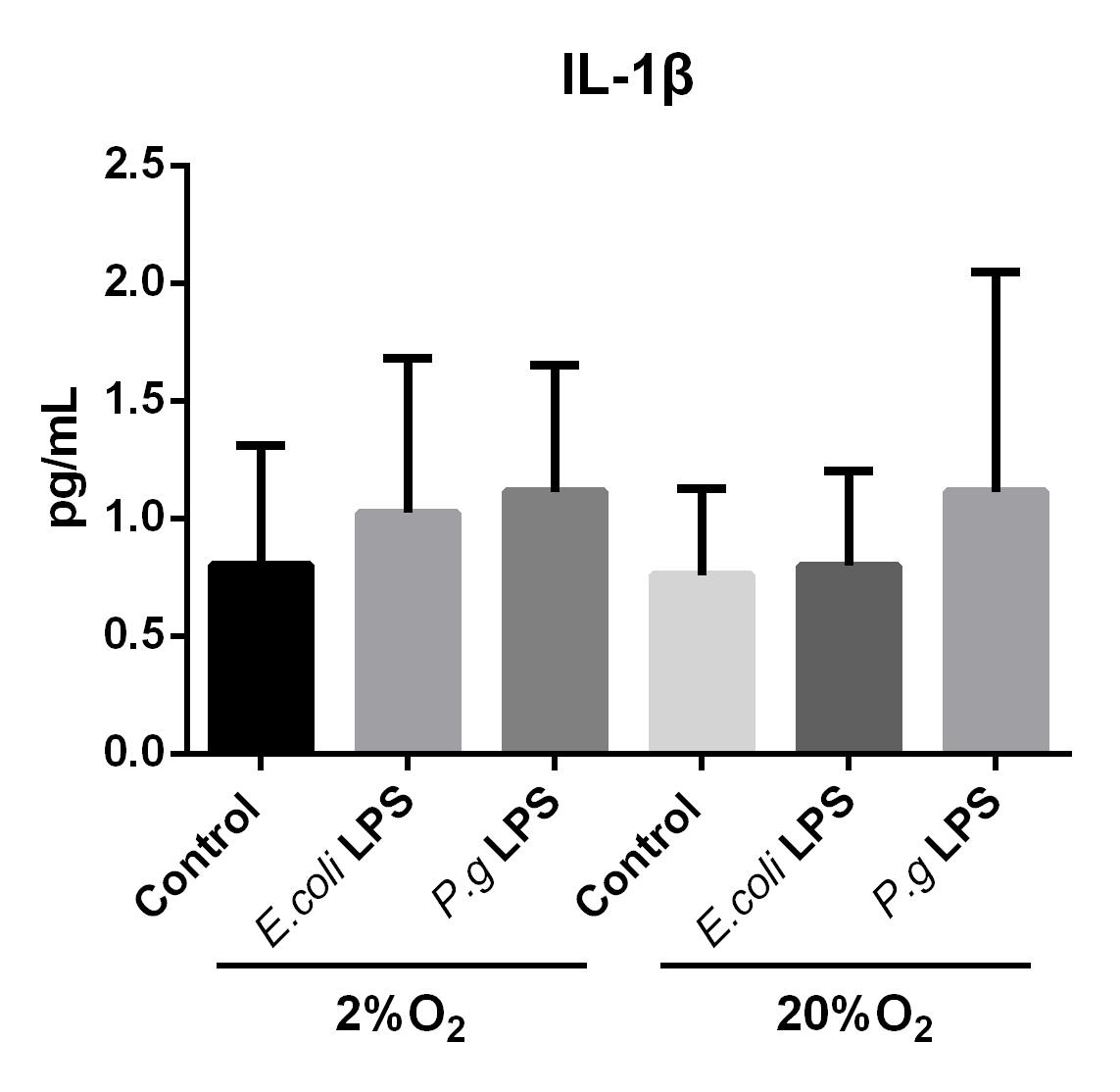

Supplement: Supplementary Figure 3 — The secretory IL-1β in cell supernatant at 24h were measured by ELISA. There was no difference among groups. [file Image3.TIF]

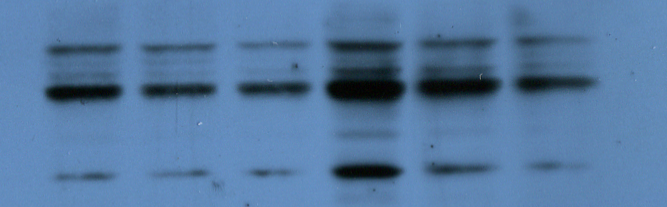

Supplement: Supplementary file 5 [file DataSheet1.ZIP › western data/caspas1-1.tif]

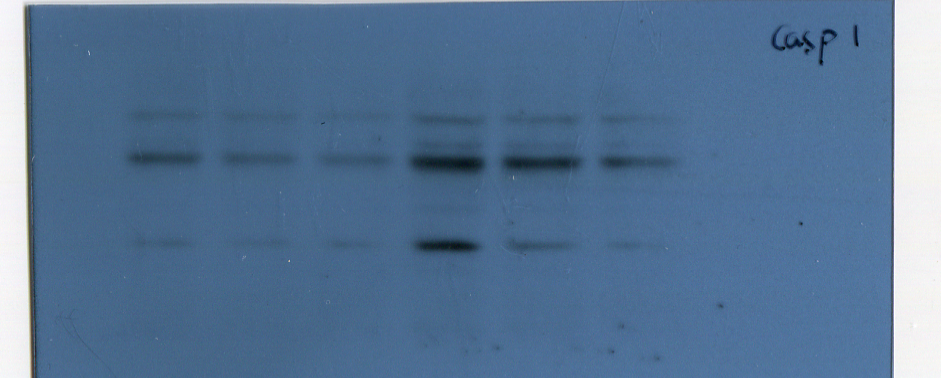

Supplement: Supplementary file 5 [file DataSheet1.ZIP › western data/caspas1-2.tif]

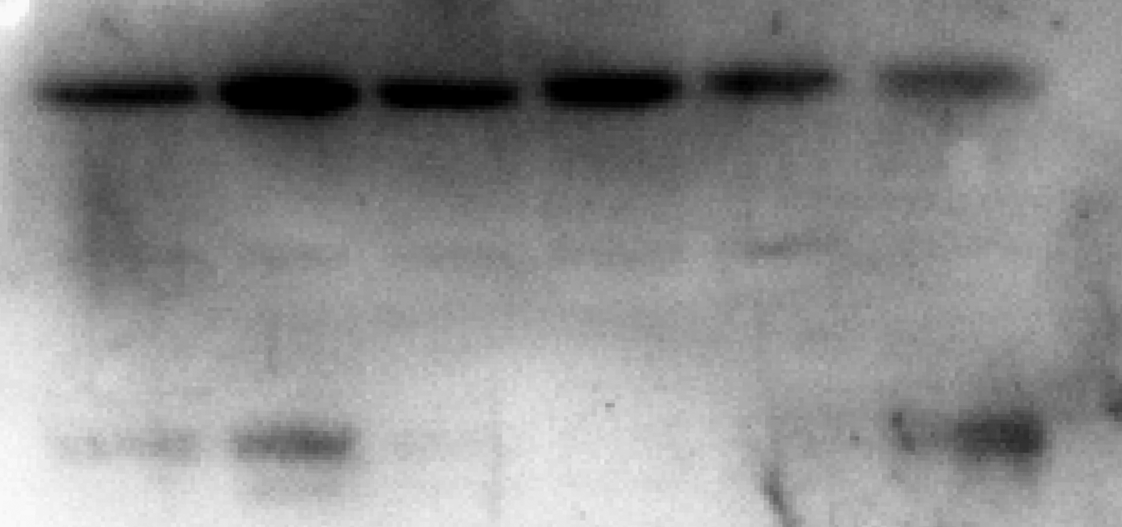

Supplement: Supplementary file 5 [file DataSheet1.ZIP › western data/caspase1-3.tif]

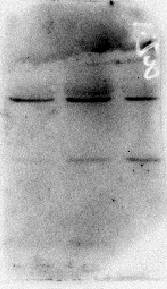

Supplement: Supplementary file 5 [file DataSheet1.ZIP › western data/caspase1-4 hypoxia.tif]

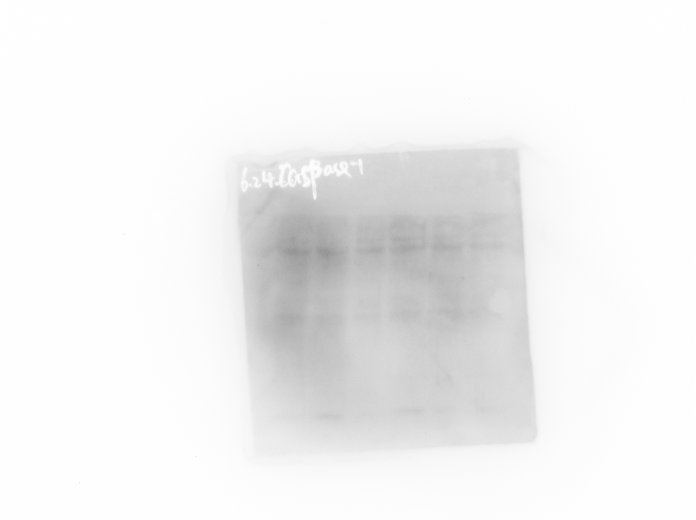

Supplement: Supplementary file 5 [file DataSheet1.ZIP › western data/caspase1-4 norm 6h-left3line.tif]

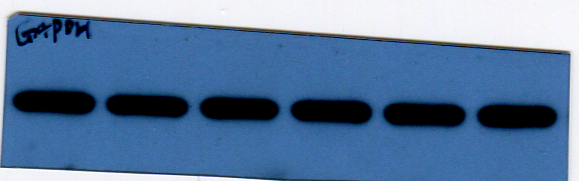

Supplement: Supplementary file 5 [file DataSheet1.ZIP › western data/gap-1.tif]

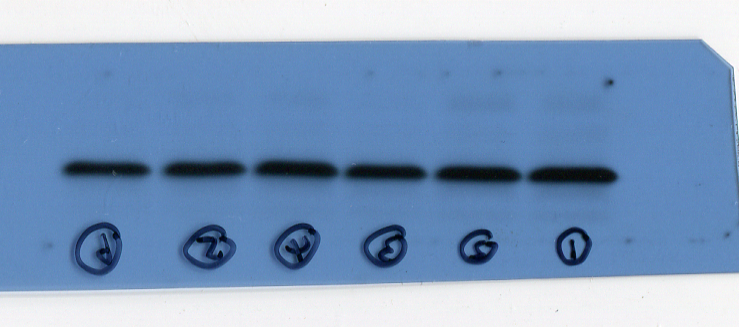

Supplement: Supplementary file 5 [file DataSheet1.ZIP › western data/gap-2 (2).tif]

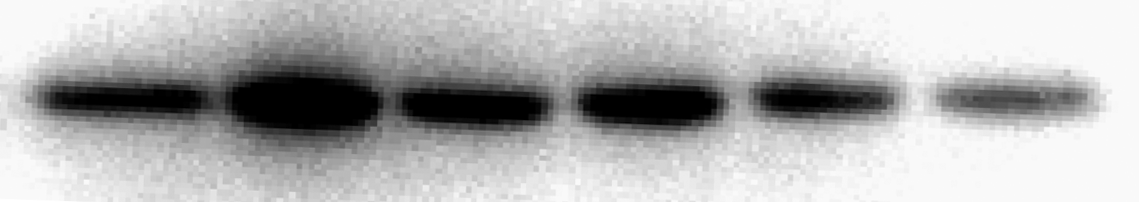

Supplement: Supplementary file 5 [file DataSheet1.ZIP › western data/gap-3-caspase1-3.tif]

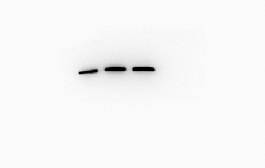

Supplement: Supplementary file 5 [file DataSheet1.ZIP › western data/gap-3-hypo.png]

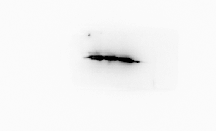

Supplement: Supplementary file 5 [file DataSheet1.ZIP › western data/gap-3-norm.png]

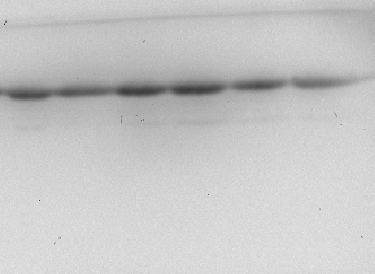

Supplement: Supplementary file 5 [file DataSheet1.ZIP › western data/gap-4.tif]

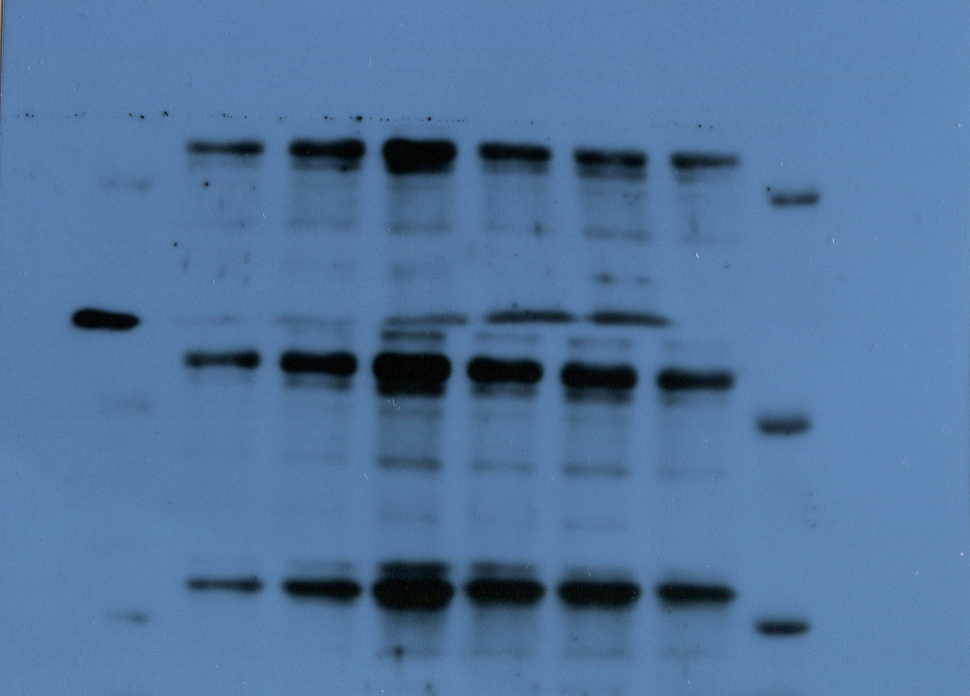

Supplement: Supplementary file 5 [file DataSheet1.ZIP › western data/il1-1-2-3.tif]

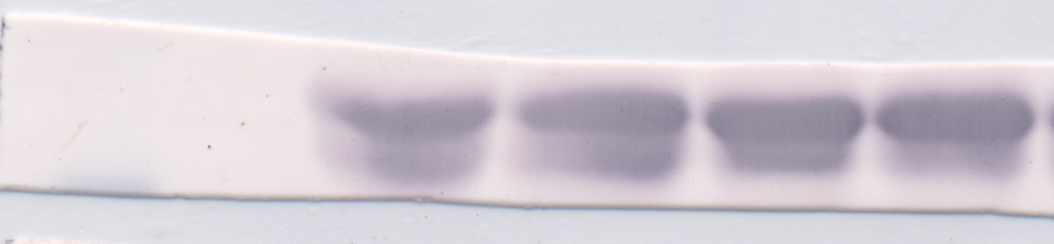

Supplement: Supplementary file 5 [file DataSheet1.ZIP › western data/mouse actin-1-2.tif]

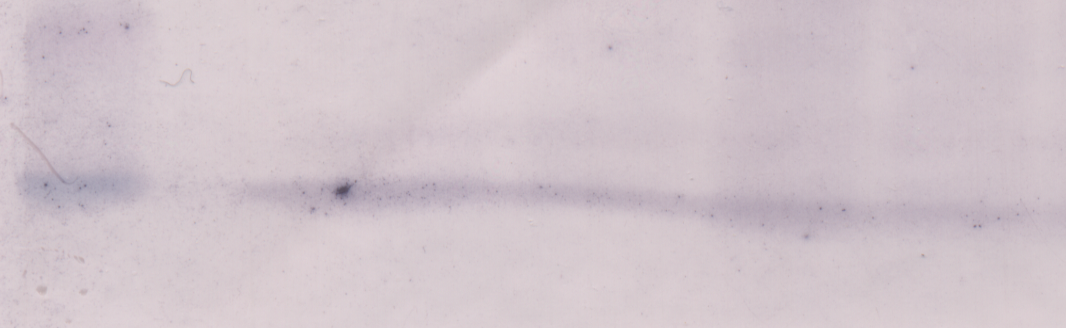

Supplement: Supplementary file 5 [file DataSheet1.ZIP › western data/mouse il1-1-2.tif]

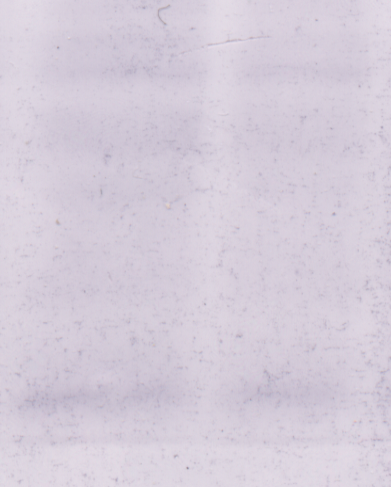

Supplement: Supplementary file 5 [file DataSheet1.ZIP › western data/mouse il1-3.tif]

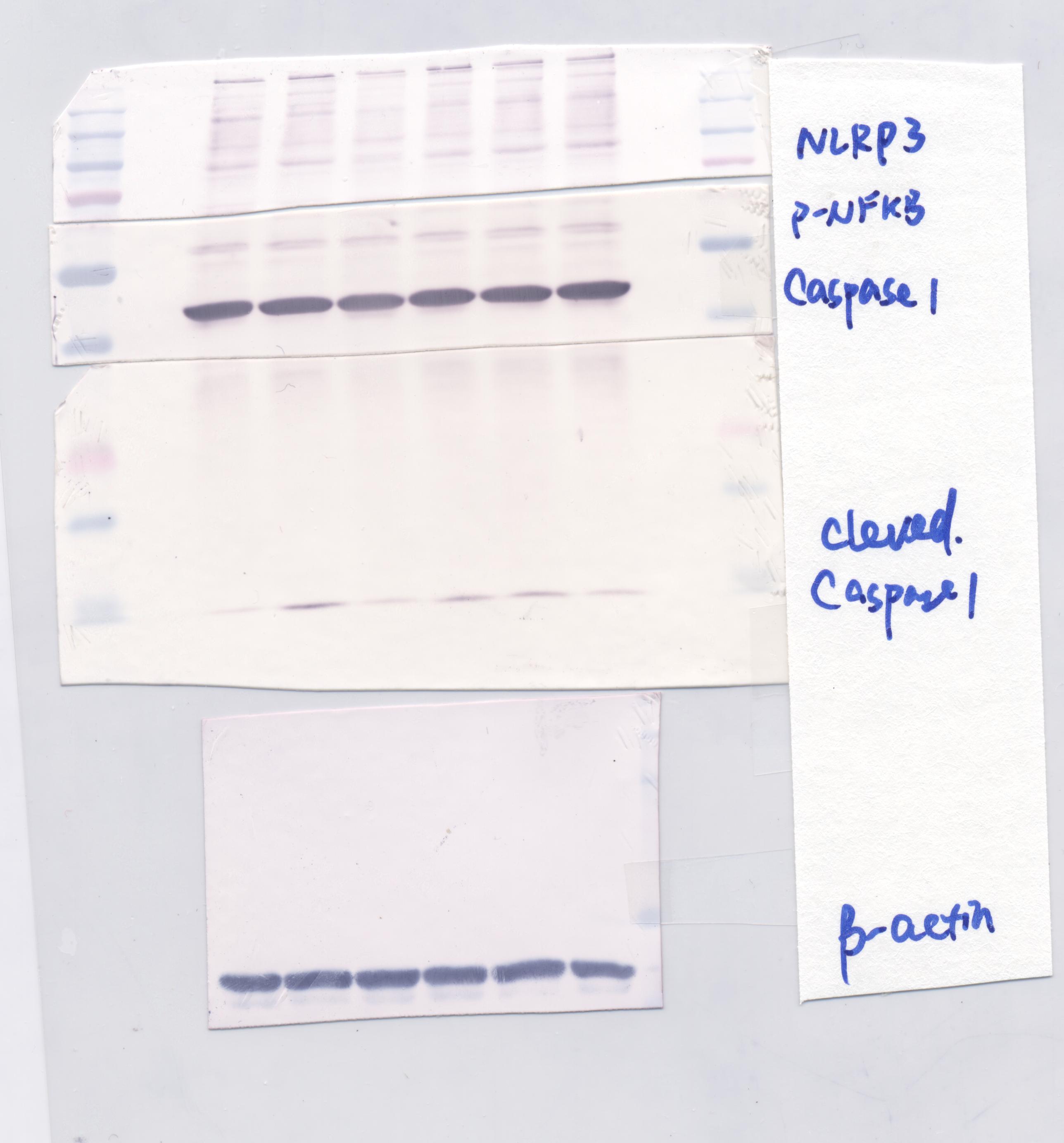

Supplement: Supplementary file 5 [file DataSheet1.ZIP › western data/mouse western.jpg]

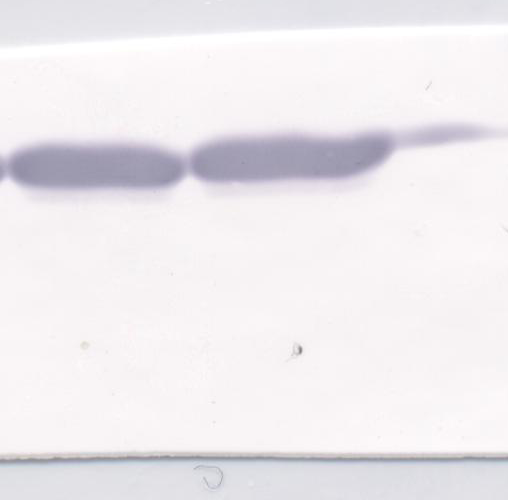

Supplement: Supplementary file 5 [file DataSheet1.ZIP › western data/mouse-actin-3.tif]

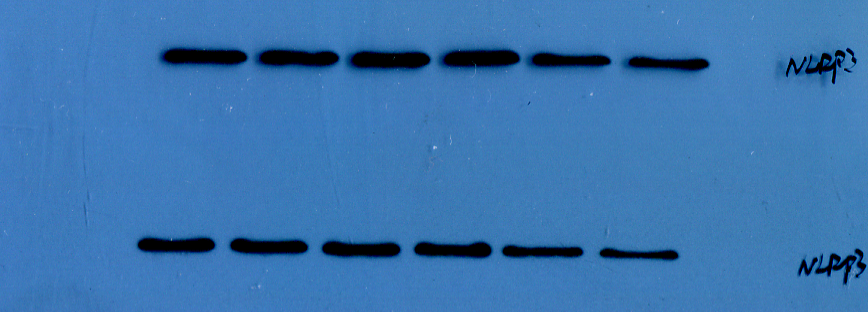

Supplement: Supplementary file 5 [file DataSheet1.ZIP › western data/nlrp3-1-2.tif]

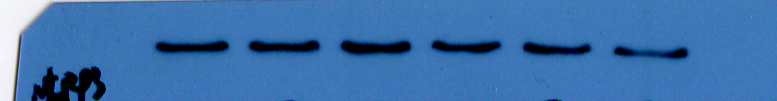

Supplement: Supplementary file 5 [file DataSheet1.ZIP › western data/nlrp3-3.tif]

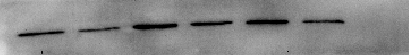

Supplement: Supplementary file 5 [file DataSheet1.ZIP › western data/nlrp3-4.png]
