# Supplementary material for: Porphyromonas gingivalis-Derived Lipopolysaccharide Combines Hypoxia to Induce Caspase-1 Activation in Periodontitis
Source: Front Cell Infect Microbiol. 2017 Nov 14;7:474. doi: 10.3389/fcimb.2017.00474 (PMC5694474; doi:10.3389/fcimb.2017.00474)
Supplement: Supplementary file 4 [file Table1.DOCX]

**Supplementary Table 1.** Information of clinical specimens

| No. | Gender | Age | Clinical diagnosis |
| --- | --- | --- | --- |
| Patient 1 | Male | 79 | Chronic periodontitis |
| Patient 2 | Female | 32 | Chronic periodontitis |
| Patient 3 | Male | 41 | Chronic periodontitis |
| Patient 4 | Female | 43 | Chronic periodontitis |
| Patient 5 | Male | 54 | Chronic periodontitis |
| Healthy donor1 | Male | 28 | - |
| Healthy donor2 | Female | 43 | - |
| Healthy donor3 | Female | 19 | - |

Diagnoses were made in accordance with clinical criteria.
